# Clinical characteristics of COVID-19 in children: a large multicenter study from Iran

**DOI:** 10.3389/fped.2024.1398106

**Published:** 2024-07-23

**Authors:** Azin Hajialibeig, Mohammad Reza Navaeifar, Amir-Hassan Bordbari, Fatemeh Hosseinzadeh, Fereshteh Rostami-Maskopaee, Mohammad Sadegh Rezai

**Affiliations:** ^1^ Pediatric Infectious Diseases Research Center, Communicable Diseases Institute, Mazandaran University of Medical Sciences, Sari, Iran; ^2^ Student Research Committee, School of Medicine, Mazandaran University of Medical Sciences, Sari, Iran

**Keywords:** SARS-CoV-2, COVID-19, MIS-C, clinical characteristics, risk factors, disease severity, ICU admission, pediatrics

## Abstract

**Introduction:**

During the COVID-19 pandemic, pediatric cases typically exhibit milder symptoms and lower mortality rates. However, the delay in pediatric vaccination poses major risks for children. This multicenter study aimed to comprehensively analyze demographic characteristics, clinical features, disease severity, and risk factors for ICU admission in Iran.

**Materials and methods:**

This observational study enrolled children aged 0–21 years with confirmed or probable COVID-19 diagnoses, referred from selected hospitals across 17 counties in Mazandaran province, Iran, between February 19 and August 14, 2021. Patients were categorized into mild, moderate, severe, or critical cases based on clinical and radiographic criteria. Data were extracted from medical records and analyzed using statistical methods. Logistic regression analysis was performed to identify factors associated with ICU admission and disease severity.

**Results:**

Among the 1,031 children included in the study, 61 were diagnosed with MIS-C. The distribution of patients by severity was 156 mild, 671 moderate, and 204 severe/critical cases. Age distribution significantly differed across severity groups (*P* < 0.001), with 55.19% aged over 5 years and 54% being male. 11.44% had underlying diseases. Fever (71.97%) was the most common symptom, followed by cough (34.43%) and dyspnea (24.83%). Within the inpatient group, 19.77% required ICU admission, with 0.91% mortality, including 3 MIS-C cases. Children with underlying diseases, gastrointestinal symptoms, and obesity had 4.16, 3.10-, and 2.17-times higher likelihood of ICU admission, respectively.

**Conclusion:**

Our study emphasized the importance of recognizing pediatric COVID-19 severity and symptoms. While fever, cough, and dyspnea prevailed, mortality rates were relatively low. However, comorbidities, obesity, and gastrointestinal symptoms linked to ICU admission, stressing early intervention. BMI also impacted disease severity and admission rate. Vaccination and targeted interventions are essential for protecting vulnerable children and easing healthcare strain.

## 1 Introduction

Coronavirus Disease 2019 (COVID-19) was declared a global pandemic by the World Health Organization (WHO) on March 11, 2020 (1) and led to approximately 7 million confirmed deaths in both pediatric and adult populations (2). The clinical presentation of COVID-19 varies, ranging from asymptomatic cases to those involving multiple organs (3). Compared to adults, children generally experience a lower incidence, milder symptoms, and reduced mortality rate (3–9). Common symptoms in children include fever and cough, with additional manifestations such as shortness of breath, sore throat, headache, dizziness, myalgia, and gastrointestinal symptoms (6, 10, 11).

Some pediatric patients, especially infants and toddlers, may require hospitalization and face a higher risk of complications and mortality (7, 10). Certain underlying medical conditions are associated with more severe symptoms and outcomes (12). Children infected with severe acute respiratory syndrome coronavirus 2 (SARS-CoV-2) are vulnerable to severe complications, including acute respiratory distress syndrome, myocarditis, acute renal and multi-organ failure, and multisystem inflammatory syndrome in children (MIS-C) (13). MIS-C represents a hyperinflammatory state linked to SARS-CoV-2 infection, posing a significant risk of morbidity and mortality (6). It's noteworthy, however, that the overall mortality rate among children remains relatively low, as indicated by a meta-analysis reporting a rate of 2.4 percent (13, 14).

In Iran, over 140,000 deaths related to COVID-19 have been reported in general population (11). However, the actual number of COVID-19 cases is likely higher (15). Numerous studies have described the clinical characteristics of children with COVID-19 in Iran since the outbreak began (7, 16–23). In a comprehensive report covering 18 months of the epidemic and 20,506 admitted patients, intensive care unit (ICU) admission, ventilator use, and total mortality rates were reported at 14.5%, 5.7%, and 2.9% of the cases, respectively (7). Another multicenter study involving 645 patients reported a mortality rate of 2.5%, primarily among individuals with comorbidities and dyspnea (18).

The vaccination rates for COVID-19 are remarkably lower in children, with many countries refraining from recommending vaccines for those under 5 years old (24, 25). Moreover, the risk of infectious disease transmission, including COVID-19, is significantly heightened within densely populated pediatric settings such as schools. In response to these concerns, this multicenter and retrospective study was conducted to analyze the demographic and clinical characteristics of children with COVID-19 in Mazandaran province. Furthermore, our study aimed to evaluate the case severity and identify risk factors influencing ICU admissions.

## 2 Materials and methods

### 2.1 Study population and setting

This multicenter observational study was performed on children aged 0–21 years with probable or confirmed COVID-19 infection diagnosis. The patients were referred from the selected referral hospitals of 17 counties of Mazandaran province, northern Iran, from 19 Feb to 14 Aug 2021.

### 2.2 Diagnostic methods

The diagnosis of COVID-19 was based on a real-time reverse transcriptase-polymerase chain reaction (RT-PCR) kit or rapid test through nasal, oropharyngeal, or nasopharyngeal swab specimens. All COVID-19 RT-PCR or rapid tests were performed in hospitals or previously determined health centers in each county. Probable cases were identified through lung CT scan findings or clinical symptoms, along with exposure to individuals confirmed to have COVID-19.

### 2.3 Severity classification

According to the WHO guidelines (26, 27; Supplementary Data Sheet 1), patients were defined into four groups of mild, moderate, severe and critical cases. The disease severity was defined as follows:

• Mild: Mild clinical symptoms with no radiographic finding compatible with pneumonia,
• Moderate: Fever, respiratory symptoms, and radiographic findings compatible with pneumonia with <30% pulmonary involvement,
• Severe: One of the following factors: respiratory distress (retraction, grunting, central cyanosis, inability to express complete sentences, etc.), tachypnea (respiratory rate ≥30/min); hypoxemia; needing supplemental oxygen and oxygen saturation (SpO2) lower than 94% in the air room; >50% pulmonary involvement,
• Critical: Acute respiratory distress syndrome (ARDS), septic shock, Multisystem inflammatory syndrome in children (MIS-C), and needing mechanical ventilation.

### 2.4 Data collection

Patients with COVID-19 were both inpatients and outpatients. Mild cases included outpatients visited by pediatricians or pediatric infectious diseases subspecialists in clinics in whom no laboratory test or chest CT scan was performed. Moderate, severe and clinical cases were inpatients admitted to mentioned hospitals and demographic and clinical characteristics and COVID-19 diagnostic test data was extracted from their medical records in addition to the COVID-19 registry system of Mazandaran University of Medical Sciences.

### 2.5 Statistical analysis

Results were presented as mean ± standard deviation (SD) or median with interquartile range (IQR) for continuous variables and frequency with percentage for categorical variables in each group. According to the Kolmogorov-Smirnov test, the distribution of quantitative variables was non-normal; So, the Kruskal-Wallis test was used for intergroup comparison. Univariate and multivariate logistic regression Cox proportional hazard models were used to determine the effective factors on disease severity during their duration of hospitalization for COVID-19 inpatients. Statistical analysis was performed by using Statistical Package for the Social Sciences (SPSS) software, version 22.0. *P*-values less than 0.05 were considered statistically significant.

## 3 Results

### 3.1 Demographics and BMI distribution

This study included 1,031 children diagnosed with COVID-19, among whom 61 were MIS-C patients. The number of mild, moderate and severe/critical patients was 156, 671, and 204, respectively. The median age of the patients was 6 years [interquartile Range (IQR): 1–14], which was 11 years in the mild group and 4 years in the moderate and severe/critical groups. The age distribution across the three groups showed a statistically significant difference (*P* < 0.001). Also, 6% of the patients were aged under 1 month, 38.8% were between 1 month to 5 years, and 55.19% were older than 5 years. Overall, 54% of the patients were male, and 58.29% resided in urban areas. 11.44% of the children had underlying diseases, with a higher prevalence in the severe/critical group (22.06%), showing a significant difference compared to the other two groups (*P* < 0.001). Body mass index (BMI) information was available for half of the patients (50.82%), among whom 17.55% were underweight, 46.18% were normal, and 36.25% were overweight/obese. It's worth noting that none of the cases had severe failure to thrive (FTT) in this study. About 50% of the patients in the severe/critical group were overweight/obese, compared to 28.57% and 35.11% in the mild and moderate groups, respectively (*P* < 0.001). 42.77% reported a history of close contact with confirmed cases with COVID-19 (Table 1).

**Table 1:** Characteristics of children with COVID-19 based on the severity of the disease.

Characteristics	Mild *n* = 156	Moderate *n* = 671	Severe/critical *n* = 204	*P*-value
Age (year) [IQR]	11 [7–15.75]	4 [1–13]	4 [1–10.21]	**<0**.**001**
Age category				** **
<1 month	3 (4.84)	32 (51.61)	27 (43.55)	**<0**.**001**
1 month-5 years	7 (1.75)	306 (76.50)	87 (21.75)	
>5 years	146 (25.66)	333 (58.52)	90 (15.82)	
Gender				** **
Male	83 (53.21)	355 (52.91)	119 (58.33)	0.386
Female	73 (46.79)	316 (47.09)	85 (41.67)	
Living place				** **
Urban	113 (72.44)	363 (71.60)	125 (76.22)	0.512
Rural	43 (27.56)	144 (28.40)	39 (23.78)	
Comorbidity	11 (7.05)	62 (9.24)	45 (22.06)	**<0**.**001**
BMI				** **
Under weight	7 (6.25)	73 (22.88)	12 (12.90)	**<0.001**
Normal	73 (65.18)	134 (42.01)	35 (37.63)	
Overweight/obesity	32 (28.57)	112 (35.11)	46 (49.46)	
Close contact with COVID-19 case	125 (80.13)	199 (29.66)	117 (57.35)	**<0**.**001**

IQR, interquartile range; BMI, body mass index.

*P*-values less than 0.05 were considered statistically significant and highlighted in bold.

A lower prevalence of patients with a normal BMI was observed in the inpatient group compared to the outpatient group (40.92% vs. 65.77%), and this difference was statistically significant (*P* < 0.001) (Table 2). Other characteristics and outcomes based on the two groups of outpatients and inpatients are presented in Supplementary Table 1.

**Table 2:** Frequency of BMI in outpatients and inpatients.

Characteristics	Outpatient	Inpatient	*P*-value
BMI			
Under weight	7 (6.31)	85 (20.58)	**<0.001**
Normal	73 (65.77)	169 (40.92)	
Overweight/obesity	31 (27.93)	159 (38.50)	

*P*-values less than 0.05 were considered statistically significant and highlighted in bold.

### 3.2 Symptoms, treatment, and outcomes

In terms of symptoms in children, fever was the most common (71.97%), followed by cough (34.43%), dyspnea (24.83%), weakness (22.79%), and nausea/vomiting (21.92%). The mild group showed a lower prevalence of respiratory distress, weakness, and skin rash, compared to others (*P* < 0.05). Approximately 25.70% and 9.50% of the patients received antibiotics and corticosteroids, respectively. The corticosteroid use was significantly higher in the severe/critical group compared to the moderate group (*P* < 0.001). 5.24% of all patients had oxygen saturation levels below 93%, and 15.42% required oxygen therapy. Within the inpatient group, 173 children (19.77%) required ICU admission, and unfortunately, 8 children (0.91%) succumbed to the illness which among them 3 patients were MIS-C (Table 3).

**Table 3:** Symptoms and outcomes of children with COVID-19 based on the severity of the disease.

Symptoms	Mild *n* = 156	Moderate *n* = 671	Severe/critical *n* = 204	*P*-value
Respiratory symptoms				** **
Cough	73 (46.79)	219 (32.64)	63 (30.88)	**0**.**002**
Respiratory distress	9 (5.77)	155 (23.10)	92 (45.10)	**<0**.**001**
Wheezing	0	1 (0.15)	2 (0.98)	0.163
Gastrointestinal symptoms				** **
Nausea/vomiting	26 (16.67)	128 (19.08)	72 (35.29)	**<0**.**001**
Diarrhea	20 (12.82)	116 (17.29)	51 (25)	**0**.**008**
Anorexia	23 (14.74)	56 (8.35)	36 (17.65)	**<0**.**001**
Abdominal pain	18 (11.54)	36 (5.37)	27 (13.24)	**<0**.**001**
Neurological symptoms				** **
Headache	52 (33.33)	34 (5.07)	16 (7.84)	**<0**.**001**
Arthralgia	9 (5.77)	48 (7.15)	6 (2.94)	0.087
Vertigo	10 (6.41)	15 (2.24)	6 (2.94)	**0**.**023**
Anosmia	8 (5.13)	6 (0.89)	0	**<0**.**001**
Loss of consciousness	2 (1.28)	7 (1.04)	3 (1.47)	0.756
Seizure	0	6 (0.89)	5 (2.45)	0.077
Restlessness	1 (0.64)	2 (0.30)	0	0.473
General symptoms				
Fever	118 (75.64)	476 (71.94)	148 (72.55)	0.489
Chills	31 (19.87)	12 (1.79)	3 (1.47)	**<0**.**001**
Myalgia	48 (3.77)	52 (7.75)	19 (9.31)	**<0**.**001**
Sore throat	41 (26.28)	11 (1.64)	8 (3.92)	**<0**.**001**
Runny nose	0	3 (0.45)	6 (2.94)	**0**.**002**
Weakness	13 (8.33)	173 (25.78)	49 (24.02)	**<0**.**001**
Skin Rash	1 (0.64)	10 (1.49)	46 (22.55)	**<0**.**001**
Conjunctivitis	7 (4.49)	1 (0.15)	29 (14.22)	**<0**.**001**
Hypotension	1 (0.64)	1 (0.15)	3 (1.47)	**0**.**050**
Antibiotic use				** **
Yes	85 (54.49)	93 (13.84)	87 (53.05)	**<0**.**001**
No	71 (45.51)	414 (81.66)	77 (46.95)	
Corticosteroids use				** **
Yes	4 (2.56)	32 (6.31)	62 (37.80)	**<0**.**001**
No	152 (97.44)	475 (93.69)	102 (62.20)	
Oxygen saturation				** **
>93%	38 (100)	621 (96.58)	92 (74.19)	**<0**.**001**
≤93%	0	22 (3.42)	32 (25.81)	
ICU admission	0	0	173 (84.80)	**<0**.**001**
Need O_2_ therapy	9 (6.08)	83 (18.69)	67 (42.41)	**<0**.**001**
Death	0	0	8 (3.92)	**<0**.**001**

ICU, intensive care unit.

*P*-values less than 0.05 were considered statistically significant and highlighted in bold.

### 3.3 Risk factors for ICU admission

Logistic regression analysis was conducted on inpatient children diagnosed with COVID-19. Clinical factors that exhibited statistical significance with a *P*-value <0.3 in the single-factor analysis were included in the multifactorial model. The results showed that children with underlying diseases, gastrointestinal symptoms, and obesity were 4.16, 3.10 and 2.17 times more likely for ICU admission than other hospitalized children (Table 4).

**Table 4:** The effective factors on ICU admission for COVID-19 by multivariate logistic regression.

Variables	OR^a^	0.95% CI^b^	*P*-value
Male gender	1.32	0.67–2.61	0.421
Obesity	2.17	1.12–4.19	**0** **.** **021**
Comorbidity	4.61	1.70–12.48	**0**.**003**
Antibiotic use	1.35	0.48–3.82	0.565
Corticosteroids use	2.87	0.60–13.87	0.189
Oxygen saturation ≤93%	1.78	0.53–5.97	0.350
Respiratory symptoms	1.95	0.96–3.95	0.064
Gastrointestinal symptoms	3.10	1.55–6.21	**0**.**001**
Neurological symptoms	1.80	0.81–3.99	0.147

*P*-values less than 0.05 were considered statistically significant and highlighted in bold.

^a^ Odds ratio.

^b^ Confidence interval.

### 3.4 Risk factors for disease severity

The result of the Cox regression model in hospitalized children with COVID-19 has been presented in Table 5. Variables with *P* < 0.3 in single-variable analysis were entered into the multivariable model. Results revealed that the variables of ICU admission and the need for oxygen therapy increase the hazard of disease severity by 9.95 and 2.34 times, respectively (*P* < 0.001) (Table 5).

**Table 5:** Determining the effective factors on disease severity for COVID-19 inpatients, using multivariate cox regression.

Variables	HR^a^	0.95% CI^b^	*P*-value
Comorbidity	1.66	0.90–3.05	0.104
ICU admission	9.95	5.61–17.65	**<0.001**
Need O_2_ Therapy	2.34	1.48–3.69	**<0.001**
Oxygen saturation ≤93%	1.34	0.81–2.19	0.251
General symptoms	1.07	0.65–1.76	0.777
Gastrointestinal symptoms	1.14	0.73–1.80	0.561

*P*-values less than 0.05 were considered statistically significant and highlighted in bold.

^a^ Hazard ratio.

^b^ Confidence interval.

## 4 Discussion

In this study, the demographic and clinical presentations of confirmed or probable pediatric COVID-19 were reported. These patients sought medical attention over six months, coinciding with the second and third waves of the pandemic in Iran. During these waves, variants such as B.1.1.7 (Alpha in the WHO classification), B.1.351 (Beta), and B.1.617.2 (Delta) were predominant, while more recent variants like Omicron had not yet emerged. The Delta variant likely led to a higher pneumonia rate, more severe disease, and increased mortality compared to other variants. However, analysis of laboratory test results did not show significant differences between variants in children (17, 28). None of the children were vaccinated during this period, but vaccination programs had been initiated for adults.

The median age of our patients was 6 years. The majority of children with COVID-19 in our study were mild to moderate, aligning with findings from other research studies (29–33). With increasing age, the hospitalization and the severity of the disease decreased. Nevertheless, approximately half of our hospitalized patients were over 5 years old. This finding is consistent with previous studies (16, 22, 34). Male gender was slightly more prevalent (1.17:1), and its frequency was a bit higher in the severe/critical group. Similar ratios have been observed in some other studies and across newborns and adults (3, 9, 16, 34–38).

Along with other studies, fever and cough were the most prevalent symptoms in our study (4, 16, 36, 39–41). Contrary to previous reports, fever was significantly more prominent in our study (37, 42, 43). In some studies, including one involving 6,610 hospitalized children with COVID-19 in Iran, dyspnea was reported as the third common symptom, supporting our findings (4, 37, 44).

After adjusting for the impact of other factors, the presence of underlying diseases, gastrointestinal symptoms, and obesity significantly increased the risk of ICU admission by 4.16, 3.10 and 2.17 times, respectively. Notably, besides ICU admission and the need for oxygen therapy, none of the other factors emerged as a risk factor for the severity of the disease. Assessing factors influencing mortality was challenging due to the limited number of fatalities.

In our study, although the specific details of underlying medical conditions (aside from abnormal BMI) were not provided, the presence of such conditions showed a significant association with the development of severe or critical disease. Furthermore, having an underlying medical condition independently increased the risk of ICU admission by more than 4-fold. This finding aligns with some prior studies, consistently highlighting underlying medical conditions as an established risk factor (36, 45).

Obesity is a known risk factor for COVID-19 (45–47). In this study, data on height and weight were available for half of the patients, in which 36.25% had a BMI above the normal range. The majority of the mild (outpatient) cases had normal BMI. O'Neill et al. reported obesity as the most significant risk factor for hospitalization, followed by diabetes (45). A lower BMI correlated with increased hospitalization, appears to have no association with the severity of the disease. One possible explanation for the impact of a low BMI was observed in the study by Crespo et al., where circulating CD3, CD4 and B lymphocytes were found to be lower in underweight children (41).

Among hospitalized patients, the ICU admission and mortality rates were 19.77% and 0.91%, respectively. Large reports from Iran indicated a significantly higher mortality rate in children, with inpatient mortality of 2.9%–5.3% in various studies (7, 37). Moreover, ICU admission rates in these studies were 14.5% and 13%, which are lower compared to our study (7, 37). Apart from methodological differences, this variation might stem from a better understanding of the presentation and management of the new disease one year after its emergence. Moreover, a well-established referral system existed in the province, facilitating the identification and transfer of severe or critical cases to referral centers. In these centers, the threshold for ICU admission was lower, and patients were admitted to the ICU earlier if possible.

In our hospitalized patients, 61 (6.97%) were diagnosed with MIS-C, constituting 37.5% (3 individuals) of our overall mortality. In a systematic review, 11.28% of examined children exhibited symptoms consistent with MIS-C (48). However, a single-center long-term study in Mexico reported a 0.6% prevalence of MIS-C (36). Therefore, MIS-C is recognized as one of the most severe consequences/presentations of COVID-19, demanding vigilant attention from the healthcare team. In our study, 4.91% of MIS-C patients succumbed to the illness. A similar study in Iran reported a 5.99% mortality rate in children, indicating comparable findings (3).

### 4.1 Limitations

Our study faced several limitations. First, due to insufficient precision in categorizing cases based on waves, we were unable to determine which symptoms were more common in each wave. Additionally, a significant portion of cases were obtained from the registry systems, which restricted our access to laboratory analysis results and imaging data. Moreover, the predominance of moderate and severe/critical patients in our study, stemming from limited participation among mild cases, compromised our ability to assess prevalence accurately. These limitations should be considered when interpreting the findings of our study.

## 5 Conclusion

In conclusion, our study emphasized the critical significance of understanding the severity and clinical presentation of pediatric patients with COVID-19, shedding light on key symptoms and risk factors for severe illness. While fever, cough, and dyspnea remained prevalent symptoms, our findings suggested a relatively low mortality rate among pediatric patients. However, underlying comorbidities, obesity, and gastrointestinal symptoms independently correlated with ICU admission, emphasizing the need for early intervention and monitoring. In addition, our analysis highlighted the role of BMI in disease severity and hospital admission. Moving forward, vaccination efforts and targeted interventions are important in protecting vulnerable pediatric populations and reducing the burden on healthcare systems.

## Data Availability

The raw data supporting the conclusions of this article will be made available by the authors, without undue reservation.

## Supplementary material

Supplementary Table 1 Clinical characteristics and outcomes of children with COVID-19 in two groups of outpatients and inpatients.

Supplementary Data Sheet 1 Critical preparedness, readiness and response actions for COVID-19: interim guidance published by World Health Organization in 2020.
