# Supplementary material for: Clinical characteristics of COVID-19 in children: a large multicenter study from Iran
Source: Front Pediatr. 2024 Jul 23;12:1398106. doi: 10.3389/fped.2024.1398106 (PMC11300238; doi:10.3389/fped.2024.1398106)
Supplement: Supplementary Table 1 — Clinical characteristics and outcomes of children with COVID-19 in two groups of outpatients and inpatients. [file Table1.pdf]

Supplementary Material

Supplementary Table 1: Clinical characteristics and outcomes of children with COVID-19 in two groups of outpatients and inpatients.

Characteristics	Outpatient	Inpatient	P-value
**Age (Year) [IQR]**	11 [7-15.75]	4 [1-12]	**<0.001**
**Gender**			
Male	83 (53.21)	474 (54.17)	0.823
Female	73 (46.79)	401 (45.83)	
**Living place**			
Urban	113 (72.44)	488 (72.73)	1.000
Rural	43 (27.56)	183 (27.27)	
**BMI**			
Under weight	7 (6.31)	85 (20.58)	
Normal	73 (65.77)	169 (40.92)	**<0.001**
Overweight/obesity	31 (27.93)	159 (38.50)	
**Comorbidity**	11 (7.05)	107 (12.23)	0.061
**Symptoms**			
**Respiratory symptoms**			
Cough	73 (46.79)	282 (32.23)	**<0.001**
Respiratory Distress	9 (5.77)	247 (28.23)	**<0.001**
Wheezing	1 (0.64)	2 (0.23)	0.389
**Gastrointestinal symptoms**			
Nausea/Vomiting	26 (16.67)	200 (22.86)	0.085
Diarrhea	20 (12.82)	167 (19.09)	0.061
Anorexia	24 (15.38)	91 (10.40)	0.068
Abdominal pain	18 (11.54)	63 (7.20)	0.064
**Neurological symptoms**			
Headache	52 (33.33)	50 (5.71)	**<0.001**
Arthralgia	9 (5.77)	54 (6.17)	0.847
Vertigo	10 (6.41)	21 (2.40)	**0.007**
Anosmia	8 (5.13)	6 (0.69)	**<0.001**
Loss of consciousness	2(1.28)	10 (1.14)	0.881
Epileptic seizure	0	11 (1.26)	0.159
Restlessness	1(0.64)	2 (0.23)	0.389
**General symptoms**			
Fever	118 (75.64)	624 (71.31)	0.268
Chills	31 (19.87)	15 (1.71)	**<0.001**
Myalgia	48 (30.77)	71 (8.11)	**<0.001**
Sore throat	40 (25.64)	20 (2.29)	**<0.001**
Runny nose	0	9 (1.03)	0.203
Weakness	13 (8.33)	222 (25.37)	**<0.001**

Skin Rash	1 (0.64)	56 (6.40)	**0.004**
Conjunctivitis	7 (4.49)	30 (3.43)	0.513
Hypotension	1 (0.64)	4 (0.46)	0.560
**Antibiotic use**			
Yes	86 (55.13)	179 (26.88)	**<0.001**
No	70 (44.87)	492 (73.32)	
**Corticosteroids use**			
Yes	4 (2.56)	94 (14.01)	**<0.001**
No	152 (97.44)	577 (85.99)	
**Oxygen saturation**			
>93%	38 (100)	713 (92.96)	0.090
≤93%	0	54 (7.04)	
**ICU admission**	0	173 (19.77)	**<0.001**
**Need O_2 Therapy**	9 (6.08)	150 (24.92)	**<0.001**
**Death**	0	8 (0.91)	-
